# Biofilm Production by Carbapenem-Resistant *Klebsiella pneumoniae* Significantly Increases the Risk of Death in Oncological Patients

**DOI:** 10.3389/fcimb.2020.561741

**Published:** 2020-12-10

**Authors:** Enea Gino Di Domenico, Ilaria Cavallo, Francesca Sivori, Francesco Marchesi, Grazia Prignano, Fulvia Pimpinelli, Isabella Sperduti, Lorella Pelagalli, Fabiola Di Salvo, Ilaria Celesti, Silvia Paluzzi, Carmelina Pronesti, Tatiana Koudriavtseva, Fiorentina Ascenzioni, Luigi Toma, Assunta De Luca, Andrea Mengarelli, Fabrizio Ensoli

**Affiliations:** ^1^ Microbiology and Virology, San Gallicano Dermatological Institute IRCCS, Rome, Italy; ^2^ Hematology and Stem Cell Transplant Unit, IRCCS Regina Elena National Cancer Institute, Rome, Italy; ^3^ Biostatistical Unit—Clinical Trials Center, IRCCS Regina Elena National Cancer Institute, Rome, Italy; ^4^ Anesthesiology, IRCCS Regina Elena National Cancer Institute, Rome, Italy; ^5^ Hospital Infection Control Committee, Istituti Fisioterapici Ospitalieri—IFO, Rome, Italy; ^6^ Department of Clinical Experimental Oncology, IRCCS Regina Elena National Cancer Institute, Rome, Italy; ^7^ Department of Biology and Biotechnology C. Darwin, Sapienza, University of Rome Sapienza, Rome, Italy; ^8^ Department of Research, Advanced Diagnostics, and Technological Innovation, Translational Research Area, IRCCS Regina Elena National Cancer Institute, Rome, Italy; ^9^ Quality, Accreditation and Risk Management Unit, Istituti Fisioterapici Ospitalieri—IFO, Rome, Italy

**Keywords:** biofilm, *Klebsiella*, carbapenem, skin colonization, cancer

## Abstract

Carbapenem-resistant *Klebsiella pneumoniae* (CRKP) is a prominent cause of nosocomial infections associated with high rates of morbidity and mortality, particularly in oncological patients. The hypermucoviscous (HMV) phenotype and biofilm production are key factors for CRKP colonization and persistence in the host. This study aims at exploring the impact of CRKP virulence factors on morbidity and mortality in oncological patients. A total of 86 CRKP were collected between January 2015 and December 2019. Carbapenem resistance-associated genes, antibiotic susceptibility, the HMV phenotype, and biofilm production were evaluated. The median age of the patients was 71 years (range 40–96 years). Clinically infected patients were 53 (61.6%), while CRKP colonized individuals were 33 (38.4%). The most common infectious manifestations were sepsis (43.4%) and pneumonia (18.9%), while rectal surveillance swabs were the most common site of CRKP isolation (81.8%) in colonized patients. The leading mechanism of carbapenem resistance was sustained by the KPC gene (96.5%), followed by OXA-48 (2.3%) and VIM (1.2%). Phenotypic CRKP characterization indicated that 55.8% of the isolates were strong biofilm-producers equally distributed between infected (54.2%) and colonized (45.8%) patients. The HMV phenotype was found in 22.1% of the isolates, which showed a significant (P<0.0001) decrease in biofilm production as compared to non-HMV strains. The overall mortality rate calculated on the group of infected patients was 35.8%. In univariate analysis, pneumoniae significantly correlated with death (OR 5.09; CI 95% 1.08–24.02; P=0.04). The non-HMV phenotype (OR 4.67; CI 95% 1.13–19.24; P=0.03) and strong biofilm-producing strains (OR 5.04; CI95% 1.39–18.25; P=0.01) were also associated with increased CRKP infection-related mortality. Notably, the multivariate analysis showed that infection with strong biofilm-producing CRKP was an independent predictor of mortality (OR 6.30; CI 95% 1.392–18.248; P=0.004). CRKP infection presents a high risk of death among oncological patients, particularly when pneumoniae and sepsis are present. In infected patients, the presence of strong biofilm-producing CRKP significantly increases the risk of death. Thus, the assessment of biofilm production may provide a key element in supporting the clinical management of high-risk oncological patients with CRKP infection.

## Introduction

*Klebsiella pneumoniae* is a major human pathogen with mortality rates up to 50%, particularly in immune-compromised individuals (Kanj and Kanafani, 2011; David et al., 2019). It causes a broad spectrum of diseases including pneumonia, urinary tract infections, bloodstream infections, skin and soft tissue infections (Melot et al., 2015; Pitout et al., 2015; Paczosa and Mecsas, 2016; David et al., 2019). Carbapenems are often considered the last line therapy for the treatment of multidrug-resistant *K. pneumoniae*  (Tzouvelekis et al., 2012; David et al., 2019). However, global surveillance studies indicate that a significant fraction of nosocomial *K. pneumoniae *isolates display extended-spectrum β-lactamases (ESBLs) and carbapenemases activities (Molton et al., 2013; Morrissey et al., 2013; Pitout et al., 2015; Brescini et al., 2019). The endemic distribution of carbapenem-resistant *K. pneumoniae* (CRKP) has been reported worldwide (Munoz-Price et al., 2013). In European countries, the population-weighted mean percentage of CRKP is 7.2%. Greece, Italy, and Romania had the highest rates of CRKP as compared to the rest of Europe (Cassini et al., 2019). Dissemination of CRKP is primarily sustained by the horizontal transfer of carbapenemase genes on mobile elements (Mathers et al., 2011; Martin et al., 2017; Partridge et al., 2018). *K. pneumoniae* carbapenemase (KPC), imipenemase metallo β-lactamase (IMP), New Delhi metallo β-lactamase (NDM), Verona integron metallo β-lactamase (VIM), and oxacillinase-48 (OXA-48) are the most common carbapenemases in CRKP (Meletis, 2016; Partridge et al., 2018). Treatment for CRKP infections is often limited to colistin, which represents, in many cases, a last-resort option due to its nephrotoxicity and neurotoxicity (Karaiskos et al., 2017). More recently, novel combinations of β-lactam- β-lactamase inhibitors, such as ceftazidime-avibactam and meropenem-vaborbactam, have been found effective against CRKP producing KPC-type and OXA-48-like enzymes, but not for those strains producing metallo carbapenemases (Bassetti et al., 2018).

The production of capsular polysaccharide is the prominent virulence factor of *K. pneumoniae* that allow this bacterium to overcome innate host immunity (Zhang et al., 2016). Currently, more than 130 different capsule types have been recognized for *Klebsiella* (Follador et al., 2016). A recent study demonstrated that *K. pneumoniae* can enhance its pathogenicity by adopting two opposing strategies based on the capsule biosynthesis. The first is related to hypercapsule production, which confers phagocytosis resistance, enhanced dissemination, and higher mortality in animal models (Ernst et al., 2020). Alternatively, *K. pneumoniae* can acquire mutations impairing capsule production, thus allowing enhanced epithelial cell invasion, increased persistence in urinary tract infections, and biofilm formation (Ernst et al., 2020). Hypervirulent strains of *K. pneumoniae* can be identified by a hypermucoviscous (HMV) phenotype on agar plates, as a result of a positive string test (Compain et al., 2014). HMV subtypes, initially described in 1986, are characterized by increased production of a capsular substance compared with classic *K. pneumoniae*, which confers a HMV phenotype (Casanova et al., 1989). Mutations in genes reducing capsule production affect the HMV phenotype and correlate with a substantial reduction in virulence when tested in mice (Walker and Miller, 2020). Thus, the HMV phenotype is directly linked with the amount of capsule production. However, a recent study demonstrated that a mutation in a gene encoding a transcriptional regulator of the mucoid phenotype (RmpC) reduces capsule production but does not affect the HMV phenotype (Walker et al., 2019). This finding suggests that HMV is dependent on the presence of the capsule, but HMV and capsule have to be considered independently (Walker and Miller, 2020). HMV, isolates showed an increased ability to cause both severe community-acquired infections such as pneumonia, liver abscesses, and meningitis in young, healthy individuals, and healthcare-associated invasive infections (Fang et al., 2007; Turton et al., 2010; Decre et al., 2011; Liu and Guo, 2019). Most HMV *K pneumoniae *strains have been related to the capsular type K1, and, in a lower fraction, with the serotype K2 (Alcantar-Curiel and Giron, 2015; Gu et al., 2018; Cubero et al., 2019) both reported as antibiotic-sensitive (Yeh et al., 2007; Gu et al., 2018). However, in recent years, carbapenem-resistant HMV strains have been reported worldwide (Gu et al., 2018; Huang et al., 2018; Lev et al., 2018; Ferreira et al., 2019; Wang et al., 2020).

Biofilm production is also important to the virulence of *K. pneumoniae* because the biofilm matrix facilitates the transfer of antibiotic-resistance mobile elements while physically protecting bacteria, thus increasing microbial tolerance to antibiotics, bacterial persistence, and dissemination (Clegg and Murphy, 2016; Ribeiro et al., 2016; Cubero et al., 2019). Biofilm eradication requires high antimicrobial concentrations, which are often impossible to achieve due to drug-related toxicity. Thus, relapses are frequent even after targeted and prolonged therapies (Clegg and Murphy, 2016; Di Domenico et al., 2019). Despite its role in microbial virulence, biofilm is not routinely assessed in clinical microbiology, and diagnosis of biofilm-related infection, in most cases, can only be presumed based on clinical signs and symptoms (Di Domenico et al., 2016).

This study analyzes the impact of different CRKP virulence determinants to assess their predictivity in supporting clinical decision-making in high-risk oncological patients.

## Materials and Methods

This retrospective study was performed at the San Gallicano Dermatological Institute and Regina Elena National Cancer Institute, Rome, Italy, between January 2015 and December 2019.

The Central Ethics Committee I.R.C.C.S. Lazio, approved the study (Prot. CE/1016/15—15 December 2015, trials registry N. 730/15).

### Microbiology

The samples were collected from a total of 86 oncological patients colonized or infected with CRKP. Bacterial identification was performed by matrix-assisted laser desorption/ionization-time of flight mass spectrometry (MALDI-TOF MS) system (Bruker Daltonik, Bremen, Germany). The antimicrobial susceptibility was assessed by the VITEK^®^ 2 system (bioMérieux, Marcy l’Étoile, France) (Lucarelli et al., 2017). Susceptibility for colistin and ceftazidime/avibactam was determined by the Sensititre broth microdilution method (Thermo Scientific, New Jersey, USA), and results were interpreted according to the European Committee on Antimicrobial Susceptibility Testing (EUCAST) clinical breakpoints (http://www.eucast.org/clinical_breakpoints). The presence of *bla*_KPC_, *bla*_VIM_, *bla*_OXA-48_, *bla*_IMP-1_, *bla*_NDM_ types was determined by the Cepheid Xpert^®^ Carba-R assay and the GeneXpert^®^ device (Cepheid, Sunnyvale, USA).

### Biofilm Formation

Biofilm production was assessed by the clinical BioFilm Ring Test (cBRT) (Biofilm Control, Saint Beauzire, France), as described in Di Domenico et al., 2016. Briefly, an overnight culture of *K. pneumoniae* grown on a blood agar plate was used to inoculate 2 ml of 0.45% saline solution to 1.0 ± 0.3 McFarland turbidity standard. The bacterial suspension was used to inoculate a 96-well polystyrene plate with 200 μl/well. The test was performed using the toner solution (TON004) containing magnetic beads 1% (*v*/*v*) mixed in the Brain Heart Infusion medium. Ten-fold serial dilutions were performed in a volume of 200 μl BHI/TON mix.* K. pneumoniae* ATCC700603 and *K. pneumoniae *ATCC 13883 were included in each plate as standard reference and internal control. After 5 h of incubation at 37°C in a static condition, wells were covered with contrast liquid, placed for 1 min on the block carrying 96 mini-magnets, and scanned with a plate reader (Pack BIOFILM, Biofilm Control, Saint Beauzire, France). The adhesion strength of each strain was expressed as BioFilm Index (BFI). Each* K. pneumoniae* strain was classified as weak moderate and high biofilm producers (Di Domenico et al., 2016; Di Domenico et al., 2017). Besides, moderate and high biofilm producers were grouped and classified as strong biofilm producers (Di Domenico et al., 2019). Each *K. pneumoniae* isolate was analyzed in duplicate, and experiments were repeated three times.

### String Test

The HMV phenotype of the CRKP isolates was revealed by the string test as described previously (Zhan et al., 2017; Liu et al., 2019).

### Sedimentation Assay

Overnight cultures were pelleted by centrifugation at 9,000×g and resuspended in PBS to an OD600 of 1. The suspensions were centrifuged at 1,000×g for 5 min, and the OD600 of the supernatants was measured. Readings were normalized to the OD600 of the strains before centrifugation (Bachman et al., 2015; Walker et al., 2019).

### Statistics

Continuous variables were compared by Student’s t-test for normally distributed variables and the Mann-Whitney U test for non-normally distributed variables. Categorical variables were evaluated using the χ2 or two-tailed Fisher’s exact test. Univariate and multivariate analyses were carried by a logistic regression model to identify independent risk factors for 30-days mortality. Statistical analyses were carried out using IBM SPSS v.21 statistics software.

## Results

From January 2015 to December 2019, 86 consecutive patients infected or colonized with CRKP were included in the study. Patients’ demographic and clinical characteristics are described in **Table 1**. The most represented underlying malignancy was hepato-bilio-pancreatic cancer (27.9%), urinary tract cancer (24.4%), hematologic malignancy (12.8%), and gastrointestinal cancer (12.8%) (**Table 1**). Infected patients were 61.6% (N53), while colonized patients accounted for 38.4% (N33). A concomitant fungal infection was detected in 5.8% (N5) of patients. Among infected patients, the most frequent manifestation caused by CRKP was sepsis (N23; 43.4%) followed by pneumoniae (N10; 18.9%), urinary tract infections (N7; 13.2%) and intra-abdominal infection (N5; 9.4%). CRKP caused 9 cases of catheter-related bloodstream infections and one case of catheter-acquired urinary infection. Among colonized patients, rectal surveillance swabs (RSS) were the most common site of CRKP isolation (N27; 81.8%) followed by urine samples (N4; 12.1%).

**Table 1:** Demographic and clinical characteristics of patients at enrollment.

Clinical Characteristics	N	%
Female	46	53.5
Male	40	46.5
Median age (range)	71	40–96
Primary Cancer		
Hepato-bilio-pancreatic cancers	24	27.9
Urinary tract cancers	21	24.4
Hematologic malignancies	11	12.8
Gastro-intestinal cancers	11	12.8
Others	19	22.1
Infected patients	53	61.6
Sepsis	23	43.4
Pneumoniae	10	18.9
Urinary tract infections	7	13.2
Intra-abdominal infection	5	9.4
Other	8	15.1
Colonized patients	33	38.4
Rectal swab	27	81.8
Urine	4	12.1
Other	2	6.1
Genotipic characterization		
KPC	83	96.5
OXA-48	2	2.3
VIM	1	1.2
NDM	0	0
IMP	0	0
Phenotype		
HMV	19	22.1
Non-HMV	67	77.9
Biofilm Production		
Weak	38	44.2
Strong	48	55.8
Clinical outcome		
Infection-related mortality	19	35.8

KPC, Klebsiella pneumoniae carbapenemase; IMP, imipenemase metallo β-lactamase; NDM, New Delhi metallo β-lactamase; VIM, Verona integron metallo β-lactamase; OXA-48, oxacillinase-48. Hypermucoviscous (HMV) phenotype.

Based on genotypic characterization, the leading mechanism of carbapenem resistance was related to the KPC gene (N83, 96.5%), followed by OXA-48 (N2, 2.3%) and VIM (N1, 1.2%). None of the strains analyzed were positive for the class B metallo-β-lactamases IMP and NDM. The OXA-48 and VIM were only isolated from RSS in colonized patients, while all CRKP from infected patients were KPC-producing *K. pneumoniae* strains. The antimicrobial susceptibility profile confirmed that almost all the CRKP strains were resistant to three carbapenems with a high level of resistance to all tested beta-lactams (**Table 2**). Among the CRKP strains, 10.5% (N9) were also resistant to colistin. Notably, only one strain were found resistant to ceftazidime-avibactam. The only CRKP isolate resistant to ceftazidime-avibactam was the VIM-positive strain. In the colistin-resistant group, seven strains were isolated from infected patients and two from colonized individuals. Among aminoglycosides, 25.6% (N22) of CRKP strains were susceptible to amikacin, and 17.4% (N15) were susceptible to gentamycin. Trimethoprim/sulfamethoxazole-susceptible isolates were 23.3% (N20), while fosfomycin and tigecycline were below the breakpoints in only 13.9% (N12) and 12.7% (N11) of cases, respectively. Notably, only 2.3% (N2) of the CRKP strains were found to be susceptible to ciprofloxacin.

**Table 2:** Antibiotic susceptibility profile of carbapenem-resistant *K. pneumoniae* clinical isolates.

Antibiotic	N	%
Amikacin	22	25.6
Amoxicillin/clavulanic acid	1	1.2
Cefepime	0	0
Cefotaxime	0	0
Ceftazidime	0	0
Ceftazidime/avibactam	85	98.8
Ciprofloxacin	2	2.3
Colistin	77	89.5
Ertapenem	1	1.2
Fosfomycin	12	13.9
Gentamycin	15	17.4
Imipenem	0	0
Meropenem	0	0
Piperacillin/Tazobactam	0	0
Tigecycline	11	12.7
TMP-SMX	20	23.3

N, number of strains susceptible for the indicated antibiotic; TMP-SMX, Trimethoprim/sulfamethoxazole.

**Figure 1:**
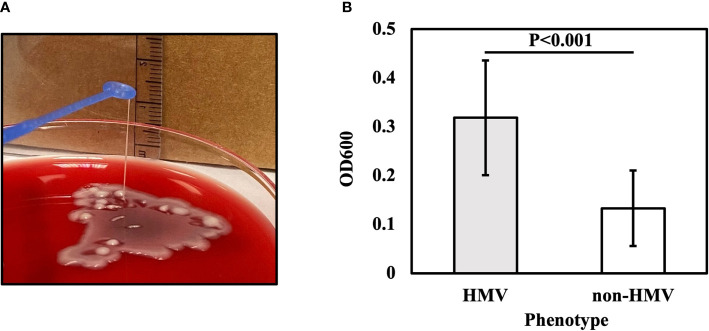
String test for identification of the HMV phenotype. A positive string test **(A)** is defined as the formation of viscous strings of >5 mm in length on an agar plate. **(B)** Sedimentation assay for HMV and non-HMV isolates.

Phenotypic CRKP characterization indicated that 22.1% (N19) of the isolates were HMV, and 77.9% (N67) were classified as non-HMV. The HMV isolates showed a positive string test result (**Figure 1A**). The median length of the string was 7 mm (ranging from 5–25 mm). The mucoviscosisty levels were determined by the sedimentation assay. HMV strains do not sediment properly during low-speed centrifugation, and the supernatant remains turbid, while the non-HMV strains produce compact pellets with clear supernatants. The turbidity of supernatant can be measured by the optical density at 600 nm (OD600) (Walker and Miller, 2020). The OD600 of HMV strains was 0.32±0.12 and, non-HMV was 0.13±0.08 (P<0.001) (**Figure 1B**).

One HMV isolate was found colistin-resistant and eight were non-HMV. Due to the low number of colistin-resistant compared to colistin-susceptible strains the difference was no statistically significant. Besides, HMV and non-HMV isolates did not show any significant association to infected or colonized patients as well as to a specific site of isolation.

**Figure 2:**
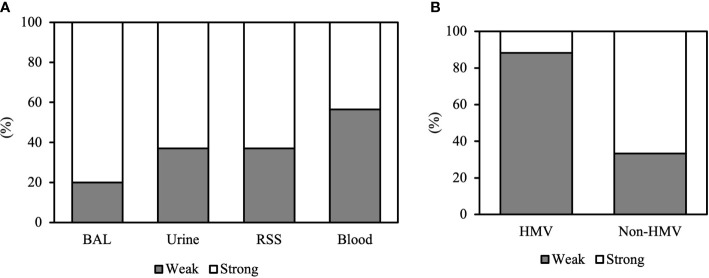
Biofilm formation of CRKP clinical isolates according to **(A)** the site of isolation and **(B)** the HMV and non-HMV phenotype.

**Figure 3:**
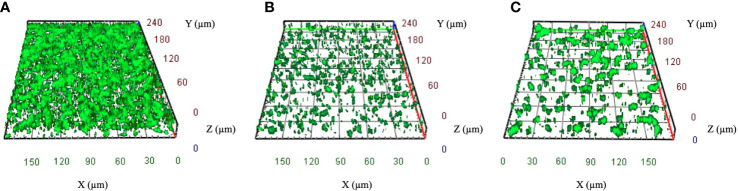
Representative confocal microscopy images of CRKP biofilms developed on polystyrene slides for 24 h at 37°C. **(A)** Strong biofilm-producing non-HMV isolates. **(B)** Weak biofilm-producing non-HMV and **(C)** Weak biofilm-producing HMV strain. Orthogonal sections displaying horizontal (z) and side views (x and y) of reconstructed 3D biofilm images are shown.

Among the 86 CRKP isolates, 55.8% (N48) were classified as strong biofilm producers, while 44.2% (N38) showed a weak production (**Table 1**). Strong biofilm-producing CRKP were equally distributed in both infected (N26) and colonized (N22) patients, while weak biofilm-producing strains were more abundant in infected (N27) as compared to colonized patients (N11). Although the level of biofilm was not significantly related to the site of isolation, strong biofilm producers were detected in 80% of BAL from patients with pneumoniae, 63% of urine samples, 63% of RSS, and 43.5% of blood cultures of septic patients (**Figure 2**). Among the colistin-resistant isolates, six were classified as strong and three as weak biofilm producers. The degree of biofilm was not significantly associated with colistin resistance. Noteworthy, biofilm production was significantly different in HMV and non-HMV strains (P=0.0002), with the former being mostly weak biofilm producers (88.2%) as compared to non-HMV (33.3%) isolates (**Figure 2**). Confocal microscopy analysis of the biofilms was performed after 24 h of incubation (**Figure 3**). The strong biofilm-producing CRKP isolates (**Figure 3A**) formed a compact 15–25 μm thick multi-layered structure. Conversely, weak biofilm-producing strains, including non-HMV (**Figure 3B**) and HMV (**Figure 3C**) isolates, were scattered over the polystyrene slide surface and no three-dimensional structure could be observed.

None of the patients colonized with CRKP died in the study period. Therefore, the 30-day mortality rate was calculated on the group of infected patients (**Table 3**). Infection-related mortality in this group was 35.8% (N19). In univariate analysis, a significantly high proportion of patients dying within 30 days had pneumoniae (OR 5.09; CI 95% 1.08–24.02; P=0.04). The presence of colistin resistance was not significantly related to increased attributable mortality in this group of patients (OR 3.08; CI 95% 0.33–28.77; P=0.32). Likewise, a concomitant fungal infection was not correlated with increased 30-day mortality (OR 4.00; CI 95% 0.33–47.73; P=0.27). Among the CRKP virulence factors, either the presence of a non-HMV phenotype (OR 4.67; CI 95% 1.13–19.24; P=0.03) or the presence of strong biofilm-producing isolates (OR 5.04; CI 95% 1.39–18.25; P=0.01) represents a significant predictive element for 30-day mortality. Further assessment of CRKP virulence factors by multivariate analysis gave a strong biofilm-producing phenotype as the only independent predictor of mortality (OR 6.30; CI 95% 1.78–19.24; P=0.004).

**Table 3:** Univariate and multivariate analyses of factors associated with for 30-day mortality in 53 patients infected with carbapenem-resistant *K. pneumoniae*.

Variables	Univariate Analysis		Multivariate Analysis	
	OR (CI 95%)	*P* value	OR (CI 95%)	*P* value
Biofilm (strong vs weak)	5.04 (1.39–18.25)	0.01	6.30 (1.78–19.24)	0.004
Colistin resistance (no vs. yes)	3.08 (0.33–28.77)	0.32	–	ns
Fungal infection (yes vs. no)	4.00 (0.33–47.73)	0.27	–	ns
Phenotype (non-HMV vs. HMV)	4.67 (1.13–19.24)	0.03	–	ns
Site (respiratory vs. other)	5.09 (1.08–24.02)	0.04	–	ns

Hypermucoviscous (HMV) phenotype.

## Discussion

Infections caused by CRKP represent a considerable clinical challenge, often burdened by a delay in the introduction of appropriate antimicrobial therapy, prolonged hospitalization, and considerable mortality rates (Gasink et al., 2009; Mouloudi et al., 2010; Freire et al., 2015; David et al., 2019). Therefore, understanding the impact of microbial infection/colonization factors on the outcome of CRKP-induced diseases may help improve patient management and prognosis.

This study analyzed data from 86 oncological patients with an infection or colonization sustained by CRKP. We found that the leading mechanism of carbapenem resistance was due to the expression of the KPC gene, present in 96.5% of the isolates, followed by OXA-48 and VIM, found in 2.3 and 1.2% of cases, respectively. These data, though from a limited group of strains, are consistent with previous epidemiological studies. Indeed, in Italy, approximately 90% of the CRKP isolates carry the KPC gene, followed by VIM (9.2%) and, in a small percentage, by OXA-48 (1.3%) (Giani et al., 2015; Navon-Venezia et al., 2017; Ripabelli et al., 2018; Di Tella et al., 2019). Different classes of carbapenemases exhibit specific functional properties and susceptibilities, which may be clinically relevant (Cassini et al., 2019). Therefore, information regarding the molecular mechanism leading to carbapenem resistance may also provide a guide in antibiotic selection and administration upon suspicion of infection (Giannella et al., 2014).

The antimicrobial susceptibility profile confirmed that almost all the CRKP strains assessed in this study were resistant to three carbapenems with high resistance levels against all the β-lactams tested (**Table 2**). Novel β-lactam/β-lactamase inhibitor combinations have been recently introduced as new treatment options against infections caused by carbapenem-resistant Enterobacteriaceae (Karaiskos et al., 2019; Sheu et al., 2019). Recent evidence indicates that ceftazidime-avibactam may represent an effective treatment for CRKP infections (Shields et al., 2016; van Duin and Bonomo, 2016; Krapp et al., 2017; Caston et al., 2017; Tumbarello et al., 2019). Indeed, ceftazidime-avibactam inhibits KPC and OXA-48 enzymes, but it is not active against the metallo-β-lactamases (ECDC EARS-NET report, 2017; Shirley, 2018; Sousa et al., 2018; Ambretti et al., 2019). Consistently with these observations, our results show that ceftazidime-avibactam is effective against KPC and OXA-48 but not against CRKP strains harboring the VIM gene (Shirley, 2018; García-Castillo et al., 2018).

Colistin is considered as an antibiotic of last resort for treating severe CRKP infections, because of increasing microbial resistance and associated toxicity (Elnahriry et al., 2016; Schwarz and Johnson, 2016; Rojas et al., 2017; Wang et al., 2018; Ghirga et al., 2020). In this study, we found a 10.5% of CRKP resistant to colistin. This result is consistent with studies performed worldwide, which confirm a colistin-resistant rate not exceeding 8.8%–13% among CRKP isolates, as assessed by broth microdilution (Goel et al., 2014; Olaitan et al., 2014; Rojas et al., 2017; Zafer et al., 2019). Previous colistin therapy was considered an independent risk factor for colistin resistance among CRKP (Giacobbe et al., 2015). In this study, the prevalence of colistin-resistant clinical CRKP isolates was relatively low. Such colistin resistance rates may indicate that infection prevention procedures and antimicrobial stewardship adopted in our institution have reduced the selective pressure, limiting the spread of colistin resistance. In our colistin-resistant group, seven strains were isolated from infected patients and two from a colonized individual. Notably, we did not observe a statistically significant difference in mortality rates between patients infected with colistin-resistant and colistin-susceptible isolates. This observation is consistent with a recent study showing that the patient’s conditions and not the presence of colistin-resistant strains have the most significant impact on the clinical outcome (Brescini et al., 2019). However, other studies pointed to a direct association between colistin-resistant strains and mortality (Giacobbe et al., 2015; Rojas et al., 2017). In particular, results from a multicenter study conducted in Italy, in which a 20% colistin resistance was found, reported a mortality rate significantly higher than that observed in patients infected with colistin-susceptible strains (Giacobbe et al., 2015).

The HMV strains represent a serious health threat, causing severe infections in both immune-compromised and healthy individuals (Shon and Russo, 2012; Shon et al., 2013; Gu et al., 2018; Liu and Guo, 2019). In critically ill patients, such as those from intensive care units, HMV *K. pneumoniae* can induce invasive infection and syndromes (Lee et al., 2010; Liu and Guo, 2019). Thus, the assessment of the HMV phenotype by the string test has been proposed as a necessary addition into the daily practice of microbiological surveillance in ICU (Hagiya et al., 2014). Globally, the prevalence of HMV strains in *K. pneumoniae* isolates is reported in the range of 17%–45% (Yu et al., 2006; Liu and Guo, 2019). The HMV strains are usually highly susceptible to antibiotics, and infections can be generally treated with success using carbapenems (Shon and Russo, 2012; Holt et al., 2015). Nevertheless, sporadic reports of isolation of carbapenemase-producing HMV strains are emerging worldwide, mostly occurring in hospitalized patients (Arena et al., 2017; Gu et al., 2018; Simner et al., 2018). In our study, CRKP-HMV strains accounted for 22.1% of the total isolates. This result is in contrast with previously reported epidemiological data showing a prevalence of about 1% (Gu et al., 2018; Simner et al., 2018; Liu and Guo, 2019). An important concern when considering the highly susceptible HMV strains is their ability to became resistant to carbapenems when subjected to a meropenem regimen (Simner et al., 2018). The carbapenem resistance in HMV appears to be maintained only in the presence of meropenem and is lost after antibiotic removal (Huang et al., 2013; Simner et al., 2018). This suggests that the presence of carbapenemase-encoding plasmids in HMV strains may someway harm bacterial fitness and is dispensable in the absence of selective pressure (Huang et al., 2013; Simner et al., 2018). Such instability may recognize several possible causes and associated factors, including the specific *K. pneumoniae* strains, the type of plasmid incompatibility groups and/or the acquisition of different carbapenemase genes (Simner et al., 2018). The exposure to multiple cycles of prolonged antibiotic treatment in our group of hospitalized patients might have exerted the selective pressure necessary to acquire and preserve carbapenemase genes in such a high number of strains. If true, this further emphasizes the judicious use of antibiotics to limit the development and spread of antibiotic resistance in hypervirulent strains of *K. pneumoniae*. Of importance, we found that non-HMV strains were associated with a significant increase in infection-related mortality. This is in contrast with a previous study describing high mortality rates caused by HMV *K. pneumoniae* strains (Shon and Russo, 2012). However, some controversies exist regarding the HMV classification and its putative virulence (Lin et al., 2011; Zhang et al., 2015). In animal models, HMV strains did not show more severe infections or higher mortality rates as compared to non-HMV (Zhang et al., 2016; Catalan-Najera et al., 2017). Besides, CRKP with an HMV phenotype were found to produce a significantly lower amount of biofilm compared to non-HMV isolates, suggesting that exopolysaccharides production has a negative impact on CRKP fitness (Cubero et al., 2019). This further confirms that the presence of the capsular polysaccharides reduces bacterial adhesion probably by the shielding of the fimbrial adhesins (Wang et al., 2015; Wang et al., 2017). However, in this reduced ability of adhesion may reside an advantage of the HMV strains. Indeed, capsule allows tighter bacterial packing, as compared to capsule-deficient cells, promoting an increased ability to disseminate to distant sites, including the lung, eye, soft tissue and central nervous system (Dzul et al., 2011; Choby et al., 2020). On the other hand, biofilm production may explain, at least in part, the association of non-HMV strains with a significant increase in infection-related mortality, since most non-HMV strains (55.8%) were strong biofilm producers, being equally distributed between infected and colonized patients. Notably, in infected patients, the presence of strong biofilm-producing CRKP significantly (P=0.01) correlated with increased mortality. Strong biofilm producers were detected in 80% of pneumonia cases, 63% of urine samples, 63% of RSS, and 43.5% of blood cultures. The fraction of strong biofilm-producing CRKP observed in this study is consistent with previous reports (Di Domenico et al., 2017; Vuotto et al., 2017; Nirwati et al., 2019; Ramos-Vivas et al., 2019). Studies directed at assessing carbapenem-susceptible *K. pneumoniae* isolated from blood, respiratory specimens, urine, and wounds, found strong biofilm producers in percentages ranging from 65% to 85% (Yang and Zhang, 2008; Hassan et al., 2011; Seifi et al., 2016; Cepas et al., 2019). The analysis of biofilm production *in vitro* showed a large variation among *K. pneumoniae* isolates according to the microenvironment, the surface where the biofilm adheres, temperature, pH, and the physicochemical characteristic of the isolate. A number of reports have pointed to an association between higher level of biofilm formation and the acquisition of a multidrug-resistant phenotype in *K. pneumoniae* (Yang and Zhang, 2008; Subramanian et al., 2012; Sanchez et al., 2013; Vuotto et al., 2017; Bocanegra-Ibarias et al., 2017; Cepas et al., 2019; Nirwati et al., 2019). In particular, an increased rate of horizontal gene transfer among bacteria growing in close contact within the biofilm matrix is deemed responsible for the rapid acquisition of antibiotic resistance, both at the single and multispecies levels (Ghigo, 2001; Madsen et al., 2012; Lebeaux et al., 2014). Despite these findings, the association between antibiotic resistance and biofilm formation is still debated (De Campos et al., 2016; Di Domenico et al., 2017; Cepas et al., 2019).

The overall CRKP infection-related mortality rate observed in the present study was 35.8%. This figure is consistent with recent studies reporting mortality rates of approximately 40% in Italy and other European countries (Hoxha et al., 2016; Xu et al., 2017; Ramos-Castañeda et al., 2018). However, geographic variations, as well as co-morbidities, should be considered. Studies in South America gave figures of 51.0% of CRKP-related mortality while in North America, a 33.2% mortality rate was reported (Rossi Gonçalves et al., 2016; GBD 2015, 2017; Xu et al., 2017). In immune-compromised patients, CRKP infection gave mortality rates higher than those observed in our study, particularly when considering patients undergoing liver transplantation (78%), or patients with hematologic malignancies and solid tumors (56%–73%) (Lübbert et al., 2013; Satlin et al., 2013; Freire et al., 2015; Ramos-Castañeda et al., 2018). We found the highest rate of mortality in patients with pneumoniae and sepsis. Similar results, in association with additional factors, including a high APACHE score, inappropriate initial antimicrobial therapy, advanced age and shock, were previously found among cancer patients infected with multidrug-resistant agents (Gasink et al., 2009; Souli et al., 2010; Zarkotou et al., 2011; Tumbarello et al., 2012; Bodro et al., 2014; Xu et al., 2017).

Assessment of these data by univariate analysis indicated that both a Non-HMV phenotype (P=0.001) and a strong biofilm-producing strain (P=0.01) are predictive of an increased CRKP infection-related mortality. Besides, multivariate analysis indicated that the presence of strong biofilm-producing CRKP strains was the only microbial factor independently associated with death (95% CI, 1.78-19.24; P=0.004) in oncological patients infected with CRKP. This result is also supported by previous study demonstrating that biofilm formation contributes to increased *K. pneumoniae* pathogenicity (Wu et al., 2011; Ernst et al., 2020). These data further support the notion that biofilm production represents a key CRKP virulence factor, which protects bacteria from physical and chemical insults, including antimicrobials, supporting microbial persistence and dissemination The effective antibiotic concentration required for biofilm eradication *in vivo* is, in most cases, impossible to reach due to drug toxicity and side effects (Ciofu et al., 2015). Therefore, the diagnosis of a biofilm-associated infection represents an area of serious concern for the clinical management of patients. The timely recognition of a strong biofilm producer, before the development of a mature biofilm matrix, may provide key decision-making elements for most appropriate targeting of either medical or surgical intervention, including type, doses, duration of antimicrobial therapy or removal of medical devices, respectively. However, conventional antimicrobial susceptibility testing performed on planktonic cells does not detect the additional resistance mechanism provided by biofilm. Thus, the introduction of reliable microbiological platforms for the diagnosis of biofilm-associated infections and the determination of biofilm-induced antibiotic tolerance represents a desirable addition in clinical microbiology.

Although bringing relevant information, this study has a few limitations. Being a retrospective study performed in a single oncological Hospital, our epidemiology findings might differ from those emerging from other experiences. Nevertheless, data from this study indicated that the mortality rate among oncological patients infected with CRKP is high (35.8%). The infection-related mortality rate did not correlate with the presence of HMV strains but, conversely, was significantly associated with non-HMV, strong biofilm-producing isolates, the latter representing an independent risk factor of death in oncological patients infected with CRKP. A more in-depth exploration of the mechanisms promoting biofilm formation in *K. pneumoniae* will help identify specific virulence markers. Nevertheless, the timely recognition of biofilm-associated infections and biofilm-induced drug tolerance still represents an unmet need in clinical microbiology.

## Data Availability Statement

The raw data supporting the conclusions of this article will be made available by the authors, without undue reservation.

## Ethics Statement

The studies involving human participants were reviewed and approved by The Central Ethics Committee I.R.C.C.S. Lazio, approved the study (Prot. CE/1016/15—15 December 2015, trials registry N. 730/15). Written informed consent for participation was not required for this study in accordance with the national legislation and the institutional requirements.

## Author Contributions

ED designed the study and wrote the paper. FM, GP, FP, IS, LP, FA, LT, AL, AM, and FE, discussed the results and implications and wrote the manuscript. ICa, FS, and TK performed the phenotypic and genomics experiments. FDS, ICe, and SP analyzed the presence of the carbapenemase genes. TK and CP collected and interpreted the clinical data. All authors contributed to the article and approved the submitted version.

## Conflict of Interest

The authors declare that the research was conducted in the absence of any commercial or financial relationships that could be construed as a potential conflict of interest.

The reviewer AA declared a shared affiliation with the authors to the handling editor at time of review.
